# The policies of *Cases Journal*

**DOI:** 10.1186/1757-1626-1-2

**Published:** 2008-05-12

**Authors:** Richard Smith

**Affiliations:** 1 BioMed Central, Middlesex House, 34-42 Cleveland Street, London W1T 4LB, UK

A journal that wants to accept not reject and to include patients as authors as much as possible

*Cases Journal *will publish any case report that is authentic, understandable, and ethical. The report doesn't have to be original or "important," and we hope eventually to publish tens of thousands of case reports a year. The accompanying editorial discusses what we hope to achieve with this new journal, and this editorial explains our editorial processes.

Case reports can eventually be submitted by anybody [1] - patients, doctors, nurses, relatives, anybody - but we are starting with simply clinicians. We are providing a template [2] that will make it easy for authors to include the information that every case report should contain (age, sex, etc), but authors don't have to use the template. We want to make it as easy as possible for authors to submit. Our staff will check for essential information, and if it's missing we'll come back to you.

It will be essential to include signed consent from the patient. Indeed, our preference is that patients will be co-authors in as many case reports as possible. It will be possible for patients but not others to use pseudonyms. We are using the same consent form as our sister journal, the *Journal of Medical Case Reports*, [3] which was in its turn derived from that of the *BMJ *[4] (In my former life as editor of the *BMJ *I became very concerned about patient consent when I realized that anonymity could not be guaranteed. [5])

There is no limit on the lengths of the reports, and if somebody can produce a report that has both the length and nuance of a Dostoevsky novel or a Freud case report then the world will be a richer place. We imagine, however, that many authors might be satisfied with 600 words. (For a rough guide you can access a report that I have written about my own case of "Beijing cough".)

Cases will be sent for peer review, but our strong bias is to publish not to reject. Reviewers will be asked only five questions:

1. Can you understand the case report?

2. Do you think that it is authentic?

3. Do you see any ethical problems?

4. Is there any missing information that you think must be added before publication? (Remember that you can add a note asking for information to be included after publication.)

5. Is it possible that this case is the first of its kind or may represent an important advance in general medical knowledge?

We will ask reviewers to answer these simple questions within hours not weeks, and we will publish their names at the end of each report. Cases will be accepted unless reviewers give us convincing reasons why they cannot be. Reviewers questions and comments will be published with the case reports. We expect that authors will want to respond to these questions and comments - starting a debate on the cases.

Case reports for which the reviewer answers yes to question 5 above will be forwarded to *Journal of Medical Case Reports *and published there if they pass its peer review. If they do not, they will be returned to *Cases Journal*. All case reports in both journals will be included in PubMed Central.

All case reports in *Cases Journal *will be open access - freely accessible immediately on publication. As there are no subscription fees to fund the journal, authors are asked to pay an article-processing charge if their manuscript is accepted for publication. Authors will not have to pay the charge themselves if their institution is a member of BioMed Central [6], which publishes *Cases Journal*.

Authors will also be able eventually to submit a question - for example: "Has anybody heard of a patient going through brain radiotherapy smelling a disgusting smell? If so, how was the patient helped?"

Having had a case report published, authors will be expected to provide follow up information. Authors will be sent a request for follow up information each year for five years. The value of the reports will be greatly increased by this follow up information, and we expect that authors will be keen to provide such information. If authors don t provide follow up then we will record that they haven't.

Readers will be able to respond to case reports - adding comments and questions. Readers will have to give their names and positions, and all those commenting will have to register on the site. We expect that authors will respond to all questions and to many comments. All of the material will be open access, meaning not only that it can be accessed for free but also that it can be reproduced without consent but with attribution.

All of the case reports will be entered into a database that readers will be able to search by things like age, sex, presentation, past medical history, comorbidity, and smoking history. We are still working on the final form of this database, but you may well be able to find a patient very like you or your patient. We will also be including in the database case reports from our sister journal and from many other sources.

This database will, we expect, become a treasure house for patients, doctors, and others - so why not send us a case or a dozen cases today?
